# The role of p16 as a biomarker in nonoropharyngeal head and neck cancer

**DOI:** 10.18632/oncotarget.26053

**Published:** 2018-09-07

**Authors:** Lucas K. Vitzthum, Loren K. Mell

**Affiliations:** Department of Radiation Medicine and Applied Sciences, University of California San Diego, La Jolla, California, USA; Center for Precision Radiation Medicine, La Jolla, California, USA

**Keywords:** p16, HPV, head and neck cancer, biomarker, non-oropharyngeal squamous cell carcinoma

The significance of p16 (INK4A) as a biomarker in nonoropharyngeal (non-OP) head and neck squamous cell carcinoma (HNSCC) is a subject of ongoing debate. In oropharyngeal HNSCC, p16 overexpression is associated with a more favorable prognosis for progression free survival (PFS) and overall survival (OS) [1]. Accordingly, p16 testing is recommended for oropharyngeal HNSCC and has been incorporated into prognostic staging [2, 3]. Increased expression of p16 is highly correlated with Human Papilloma Virus (HPV) mediated tumors of the oropharynx, as the HPV E7 oncoprotein inactivates pRb, which induces upregulation of the cyclin-depended-kinase, p16 [1,4]. In non-OP HNSCC, however, the prognostic role of p16 as well as its association with HPV is less clear.

A pathologic review of p16 expression *via* immunohistochemistry (IHC) from cooperative group trial patients found that p16 positivity in squamous cell carcinoma (SCC) of the oral cavity (OC), hypopharynx and larynx was associated with significantly improved PFS (HR = 0.6, 95% CI = 0.42 - 0.95) and OS (HR = 0.56, 95% CI = 0.42 - 0.95) [4]. Interestingly, this study also found that high risk HPV status *via in situ* hybridization (ISH) was not correlated improved with PFS or OS. p16 and HPV status were found to have a higher rate of discordance in non-OP HNSCC than in tumors of the oropharynx, where p16 is routinely used as a surrogate for HPV status [4]. Further investigation is warranted to determine the validity of p16 as a surrogate marker for HPV in non-OP HNSCC. It is also of significant interest whether expression of p16, an important cell cycle regulator, predicts for a more favorable prognosis independent of HPV status.

High risk HPV status *via* seropositivity for HPV16 E6 and E7 has also been associated with improved OS in SCC of the OC (HR = 0.45, 95% CI = 0.25 - 0.70) and larynx (HR = 0.29, 95% CI 0.25 - 0.70) [5]. Alternatively, Fakhry and colleagues evaluated a retrospective, multi-institutional cohort and found no significant association between p16 or HPV status and survival in non-OP HNSCC (HR = 0.80, 95% CI 0.54-1.18) [6]. Most recently, our group published on a cohort of HNSCC patients using the US Veterans Affairs database and found that p16 had a similar prognostic role for OS in both non-OP (HR = 0.41, 95% CI = 0.25 - 0.69) and oropharyngeal cancer (HR = 0.53, 95% CI = 0.40 - 0.71) [7]. Discrepancy between studies regarding the prognostic role of p16 in non-OP HNSCC is not fully understood.

Prevalence of p16 in non-OP HNSCC ranged from for 6-26%, 13-20% and 16-21% in the OC, larynx and hypopharynx respectively [6–8]. Decreased power due to the lower incidence of p16 in non-OP HNSCC could explain the negative findings in some trials. Inclusion criteria varied between studies; most notably, whether definitive surgical and nasopharynx patients were included [4, 6, 7, 9]. It should be noted that scoring systems for p16 positivity (≥ 70% of cells involved) were validated for oropharyngeal primaries and may need to be re-evaluated for non-OP HNSCC [4]. Some have hypothesized misclassification of primary anatomic sites or ectopic tonsillar tissue as an explanation for non-OP HPV and p16 positive tumors [4, 6]. If true, this may in fact support more widespread testing for p16 outside of the oropharynx to biologically classify tumors in addition to their anatomic classification.

In oropharyngeal cancer, where the prognostic role of p16 and HPV is more established, there is an effort to identify patients that could benefit from treatment deintensification [10]. In our recent study, p16 positive patients were found to have a decreased risk of non-cancer mortality in addition to cancer mortality [7]. Future trials should consider a patient’s cancer risk in relation to their non-cancer risk when considering treatment intensification. While p16 positive patients may have improved PFS and OS they may also have a decreased risk of non-cancer mortality, increasing their likelihood of observing a benefit from more intensive treatment.

Further research is required to understand the prognostic implications of p16 outside of the oropharynx, particularly with respect to treatment interaction and individual subsites. For HNSCC of the OC, larynx and hypopharynx, p16 has been associated with improved survival in multiple well designed studies and warrants consideration for more widespread testing [4, 7].
